# Three Years with the Army of the Potomac—A Personal Military History

**Published:** 1911-10

**Authors:** William Warren Potter

**Affiliations:** Buffalo, N. Y., Brevet Lieutenant Colonel, U. S. Volunteers; Surgeon in Charge of First Division Field Hospital Second Army Corps; Surgeon 57th Regiment New York Volunteers; Assistant Surgeon 49th Regiment New York Volunteers; Recorder Second Division Hospital Sixth Army Corps, etc., etc.


### Three Years with the Army of the Potomac—A Personal Military History

By William Warren Potter, M.D., Buffalo, N. Y., Brevet Lieutenant Colonel, U. S. Volunteers; Surgeon in Charge of First Division Field Hospital Second Army Corps; Surgeon 57th Regiment New York Volunteers; Assistant Surgeon 49th Regiment New York Volunteers; Recorder Second Division Hospital Sixth Army Corps, etc., etc.

We left our camp near New Bridge on Gaines's farm, at three o'clock Thursday morning, June 5, and marched back to the R. R. at Despatch Station, a distance of eight miles. Crossing the river on the R. R. bridge, we then moved west seven miles towards Richmond, which brought us to Golding's Farm, situate about seven miles from the rebel capital, where we halted. The rains have made the roads and fields muddy and sodden, and we bivouacked that night in a clover field where the horses would sink over hoof in a moment; and this is the place we are to stay in for a month. June 6. The whole regiment is on fatigue duty corduroying roads and building bridges, to enable the artillery and baggage trains to cross over from Dr. Gaines's, where we were, to this point where we are, not more than two or three miles, but which cost us a detour march of sixteen miles to reach. On the 8th our baggage train arrived, so we are living again. We are camped within rifle's shot of the enemy's pickets, protected from their view by simply a skirt of woods. Our brigade has a front line, and is engaged in throwing up intrenchments. Dr. Hall is some better, but not on duty. There is considerable feeling of discontent in the regiment, and some of the officers talk about resigning. I wrote my wife today, in reply to a letter from her beseeching me to come home, that I could not think of resigning in the face of the enemy, especially as such an act would subject me to disgrace forever after. Tom Dewey comes in to see me every day, and his face is real sunshine in the camp.

Monday, June 9. It is cool and comfortable, and the regiment is busy strengthening its rifle-pit front. A small redoubt has been built just at our left, and guns are being mounted in it today. It looks very much as though our Yorktown work was to be repeated here. The 20th N. Y., Colonel Vegesack, (formerly Max Weber's Regiment), joined our brigade today 1,040 strong, and is a fine looking body of men. They have been at Fort Monroe so long that they look as prim as regulars—in marked contrast to the other troops.

June 10. A cold, disageeable rain set in about midnight, and the weather today seems more like October than June. The enemy complimented us with a few shells and solid shot just at dusk last evening. While Dr. Hall, Dr. Mulford and I were standing in front of my tent, engaged in conversation, a six-pound solid shot passed over our heads, striking the ground about thirty yards in our rear; another passed over the Fort on our right, and struck near General Smith's headquarters, and several shells burst in the air twenty or thirty yards to our left. Today they began the same sort of entertainment again, but the shells all burst farther to our left, and fell a little short of the line. Wheeler's battery is stationed just at the left of our regiment, but has not yet replied. Captain Wheeler intimated to me today, however, that he should do so the next time they opened fire.

General Prim, lately in command of the Spanish forces in Mexico, paid our camp a visit last Sunday (the 8th), accompanied by General Smith, staff and cavalry escort. He was also attended by several officers of his own suite, wearing the Spanish uniform, which is much more showy than our soiled and tattered dress, presenting thereby a strong contrast. Major Johnson is improving in health, and bids fair to return to duty soon. Dr. Hall is also some better, but I am very suspicious he will be obliged to give up for a time, and possibly go North before he fully recovers.

June 11. This evening the "Rebs" paid us the further compliment of half-a-dozen shells, but without damage. One burst near the color-line, and another in a piece of woods nearby on our left. General McClellan and staff paid us a visit today, he and General Smith visiting the pickets and reconnoitering the lines together. Captain W. F. Wheeler of "Ours," not the artillery officer just mentioned, is not well, but I hope his illness will not prove serious. Today we hear that Memphis has fallen, and that the entire Mississippi is in our possession.

June 12. About noon the rebels sent us four or five shells; again at five o'clock P. M. they did the same; and yet again at sundown, as Colonel Bidwell, Lieutenant Dewey, and I were sitting in front of my quarters a shell passed directly over our heads, killing ont of the Cameron Dragoons near Hancock's Brigade; another of the Dragoons was killed on picket today. An alarm on the picket line at one o'clock last night turned out the regiment, but it proved a stampede and we went to bed in an hour, after taking a good view of the eclipse of the moon.

June 13. The regiment is in line of battle every morning at three o'clock and stands under arms until half an hour after daylight, so we don't propose to be surprised by our treacherous foe. This morning we were shelled for two or three hours, but again without damage. They must have fired 100 rounds at us, but the projectiles either fell short or went over our heads. They don't seem to get our precise range, though they come pretty close to it sometimes. General McClellan visited our lines again today, and, as usual, was greeted with great enthusiasm. General Davidson expects an attack tonight, but he frequently gets such an idea into his head, so we don't scare much under that pleasant little bit of camp news.

June 14. It was so extremely hot today that I stripped myself down to my underwear, and lay sweltering in my tent at that. Dr. Hall is not as well and has gone back to Dr. Trent's house, where a hospital has been established, just adjacent to General McClellan's headquarters. Captain Drake is also there, and I visited them both today. I can hear the bands in the enemy's camps playing quite distinctly tonight, which generally means mischief; but Lieutenant Colonel Alberger and 275 men from the 49th are on picket tonight, so we shall be quiet I think, for he keeps the line straight when he is field officer.

June 15. The heat continues so intense that I sent to the woods for young trees, and planted them around my tent to protect it from the sun's rays. This P. M. a thunder shower refreshed and cooled us somewhat; but the pickets were driven in somewhere on our left just as the storm subsided. Captain Wheeler is quite sick, though not dangerously so, I think. He has worked hard of late, having no Lieutenant to assist him, and the fatigue has been too great for his endurance. I sent him to the Trent House today, where Dr. Hall is, where he will be more comfortable, and where his recovery will be facilitated by removal from the noise and excitement of camp. Major Johnson is better, but Colonel Bidwell is not well, though he keeps on duty. Dewey is well, and as genial as ever; so is Alberger.

June 16. The weather has turned cooler. These changes produce considerable sickness among the troops, yet our regiment suffers less in this respect than some of the others in the division. Today the long expected flag, presented by the ladies of Buffalo, arrived and was committed to the custody of the regiment with ceremony. The command formed in hollow square and, in the presence of General Davidson and staff, Colonel Bidwell presented the colors to the regiment with appropriate remarks, followed by the rousing cheers of the soldiers.

Lieutenant McLean was wounded and taken prisoner two or three days ago somewhere in the vicinity of Liberty Hall, or Old Church, where he was doing out-post duty on the extreme right of the army. It appears that the bold rider, Stuart, made the circuit of the Army of the Potomac with 1,500 of his troopers, striking McLean's squadron at the point named, and riding over it pell mell, killing Mac's beautiful white mare, and wounding him in the head. We have heard today of McLean's safe arrival in Richmond.

June 17. This is a quiet day and I took advantage of the lull to visit Dr. Hall and Captain Wheeler at the Trent House, finding them both better. Captain was sitting on the veranda enjoying the refreshing breeze of a mild June afternoon, and I hitched my horse and joined him. We talked over "old times" for an hour, and he seemed better from my visit. It is rumored that the rebels intend to attack us tomorrow, in commemoration of the battle of Waterloo. We shall see. Mr. DeFort, of Company D, has been established as my valet-de-chambre, and he keeps my quarters in such fine order as to excite the envy of all my visitors, who are not a few.

June 18. The enemy attacked General Sumner's line on our left, just at night, killing six and wounding fourteen men. They sent forward two regiments and deployed their skirmishers on our pickets, but were soon driven off and the lines held as before. We are under orders to move at a moment's notice, and have so been since noon.

June 19. All is quiet along the lines, but I think, from movements and preparations which I observe all around, that something is going to be done soon. The North is impatient as I observe from the papers, but this is a great machine to manage and, with a wily enemy opposed, movements must be conducted with caution.

June 20. By official orders from General Headquarters our camp has been designated "Camp Lincoln," in compliment to our illustrious President—a fitting recognition of his greatness. Captain Wheeler is improving; Colonel Alberger is quite sick; Dr. Hall is about the same; Major Johnson is on duty again.

June 21. I visited McClellan's headquarters where I found a sutler who had plenty of good things; I bought a box of figs, and tonight Colonel Bidwell and Major Johnson have enjoyed them with me, in my tent. We are still entertained daily—sometimes two or three times a day—with the shelling by the enemy, generally, however, without damage to our regiment 9 o'clock P. M. Colonel Bidwell has just received an order to send out a company to strengthen the pickets, and Company A goes in compliance therewith.

June 22. This is the most quiet day we have had for sometime—not a shot fired within hearing of our camp. Captain Wheeler is better, and returned to duty today; Alberger is still quite sick. Lieutenant C. H. Bidwell has resigned—his papers came today. He gave me a fine canteen, with an outside pocket for knife, fork, and spoon, and two tin cases for pepper and salt.

June 23. Lieutenant Bidwell started for Buffalo today, and I sent a box of cigars home by him, which were made by one of our men, from tobacco that grew on Dr. Gaines's plantation

June 24. A great thunder shower prevailed all last night, and another came today. I never experienced anything like such severe storms of that character as we have here. The lightning was so continuous that it was quite light the most of the night, and the electricity came so near the earth as to nearly convulse my extremities several times. The rebels are building a fort in our front, which we have just discovered. Four prisoners, probably deserters, were brought through our camp today, on their way to Headquarters.

June 25. Today has been one of considerable excitement; we have been under orders to be ready to move all day, the men keeping their belts on, and extra rations and ammunition have been issued. The line on the left was advanced somewhat today, and the artillery on the right, across the river, has kept up a continuous fire all day from three batteries of thirty pounder Parrott's, apparently at the Fort which the enemy is building in our front. Dr. Hall is better; Colonel Bidwell and Major Johnson are in good spirits, while Alberger is still sick, though a little better today.

June 26. This has been a day of great excitement. About two o'clock P. M. we heard guns in the direction of Mechanicsville, about four miles away, on our extreme right; within an hour the firing so increased as to lead to the conclusion that an important battle was going on; and within another hour we heard that "Stonewall" Jackson had appeared on our right flank with 30,000 men, and that Porter, McCall, and Stoneman were giving him battle. Long and anxiously did we listen to the distant roar of the battle, the cannonading was furious, and at night-fall we began to see the shells bursting over the tree-tops. About eight o'clock P. M., a dispatch from General McClellan was read to the troops, stating that we had driven the enemy from the field. The men fell into line, began to cheer, and regiment after regiment took it up along the whole line towards our left, until it seemed as though pandemonium had broken loose. At nine o'clock another dispatch stated the enemy to be fleeing towards Richmond, our cavalry and horse-artillery in pursuit. Again the cheering began, and the bands commenced playing, keeping it up until near midnight, the first music we have been allowed since we left the White House. Our loss is supposed to be considerable, as all the ambulances belonging to our command have been ordered to the scene for duty.

June 27. Hancock's Brigade and two regiments of our own, including the 49th, were ordered out this morning to the picket line, all under the command of Hancock. I, of course, went along. We were partially concealed in a piece of woods, but could see the enemy at work on the fort I have before alluded to. We had not been out long before the 6th Maine regiment, who are experienced wood choppers, began cutting down the timber that protected our camp from the view of the enemy. Meanwhile, a battery of seventeen guns—six three-inch guns, six light twelves, and five thirty-pounder Parrott's—was put in battery behind the timber, and after it fell we were in full view. We remained out until this had been accomplished, and then went back to camp, reaching there about noon. The men stacked arms and began, at once, to cook their dinner. Our own dinner was served as usual under the Colonel's fly, and while Colonel Bidwell, Major Johnson, Captain Wheeler and myself were eating, the enemy opened upon us with a heavy fire. The shells flew over our heads thick and fast, a piece of one striking my colored servant's tent just in rear of my own. He was lying down fast asleep at the time, and it awakened him with such fright that he ran away across the field, and never came back. I never saw him afterwards, but learned that he made his way to Harrison's Landing with the army, where he took the first boat for the North. He called upon Dr. McCray, in Buffalo, on his way to Detroit where he lived. He had met the doctor the winter previous, when he visited me at Camp Griffin. To return, the regiment immediately fell into line, and soon we were ordered to the left in support of the battery which had just been erected, occupying some breast-works all the afternoon, and from this point we could plainly see the battle of Gaines's Mill, which was then going on under General Porter.

By nightfall it was plain to be seen that Porter had lost ground, but we did not then realise the full significance of his battle. We went back to our own camp again just at night, and while the men were having supper, an order came for our brigade to go to Porter's assistance. While the regiment was marching out by the flank to obey the order, the enemy opened upon us with artillery; shells began to fly all around us, one bursting in the flank of the regiment, killing and wounding several men. I dismounted and took the wounded forward into the rifle-pits for shelter, when the order for us to move was countermanded. One of the men killed was the man who made the cigars, that I sent home by Litutenant Bidwell. We remained in line under arms all Friday night, under the greatest tension of nerve and muscle, hourly expecting the enemy to attack.

June 28. The night passed without event, and this morning I rode back to the Trent House Hospital, to see about my wounded who had been sent there the night before. Here I found everything had been ordered away towards Savage's Station, Dr. Hall having gone with the rest; so I returned to camp. While I was away on this mission the rebels attacked our line with the 7th and 8th Georgia Regiments, and were driven off handsomely, leaving Colonel Lamar, of the 8th, in our hands severely wounded. Captain Theodore B. Hamilton, of the 33d was taken prisoner by the rebels in this affair. He is the eldest son of Dr. Frank Hastings Hamilton. This ended the fighting for the day, so far as we were concerned, and at night we lay in the woods, on our left, supporting Mott's battery. Colonel Bidwell, Captain Mott, Major Johnson and myself slept on the ground, in a tent which we found deserted. Our own camp was left standing, but during the night the tents were cut into ribbons, to render them useless to the enemy.

June 29. Our orders were to remain until daylight, then to retire guarding Mott's battery, which we did, and reached the Trent House about eight o'clock A. M., where we found the division drawn up in line. We then moved down along the Chickahominy, guarding the fords and bridges during the forenoon, and brought up on the field of Savage's Station about two o'clock P. M. Here we found Sumner's 2d Corps drawn up expecting the enemy. Our Division took position over in his left near the Williamsburg Road, and rested for an hour or more. Soon after four o'clock, as the enemy did not appear, we moved down the Williamsburg Road about a mile, and rested again. At five o'clock the enemy arrived and attacked Sumner at the Station, whereupon we were recalled to assist him. Brook's Vermont Brigade engaged the enemy at the opening through which the Williamsburg passes out into the field, and lost quite heavily. Some other portions of the Division also suffered, but not as severely as Brook's. These wounded were collected at a house and shop about a mile down the road from the field of action, where a temporary hospital had been established.

### A Prisoner.

About eight o'clock in the evening of the 29th, Sunday, while I was busily engaged in the care of the wounded, Dr. Brown, Franklin's Medical Director, came to tell me that the Army was in full retreat, that these wounded men were to be left behind and that, by General Franklin's order, he would detail me as one of the medical officers to remain with them. Dr. Russell of the 5th Vermont, and Dr. Sawin of one of the Vermont Regiments, were also to stay. We kept at our work most of the night, but finally, at near three o'clock A. M., laid down for a little rest, from sheer exhaustion. Meanwhile the army was passing in steady tramp until midnight, when the rear guard went by, leaving only a battery behind; and this by mistake, as I learned afterwards.

Though so very tired I yet could not sleep from the excitement of the situation, and at dawn I arose and began work. I soon heard the enemy's skirmish line advancing slowly through the woods, and presently it came to a halt on a line with the house where we were. I was examining a man's face at the time, who had some of his teeth knocked out, and had placed him in the doorway to get more light when, looking around, I saw some mounted men peering in at me. They proved to be some of General Jackson's officers, whose column was moving on this road, so I told them, briefly, by what authority I was there, and what my business was. One of them, comprehending the situation at once, remarked that he would report the matter to General Jackson, turned upon his horse to go and do so, when Jackson himself rode up. I briefly told my story to him, stating that I had no rations, and needed a guard to prevent the troops from over-running the place, and particularly to preserve the water supply. He directed that a Sergeant and twelve men be detailed as guards, committed other questions arising from our presence there to his Medical Director, and ordered the column to advance.

Sometime during the morning, about ten o'clock I should think, the head of D. H. Hill's Division halted near the Hospital, and from General Hill a pass was obtained which authorised me to visit the field of battle, in quest of any wounded who might have been overlooked. About two o'clock P. M., armed with the pass and accompanied by one of the guards, I proceeded to the field for the purpose mentioned. In going I was obliged to pass the Confederate column, which was still marching along the Williamsburg Road, en route for White Oak Swamp. They were a cherry lot, flushed with their recent victories which they vainly supposed decisive, and greeted me with many playful expressions, such as "On to Richmond," "You are on the right road," etc., etc. Their uniforms (?) were tattered, but their muskets were bright, and their cannon, though mostly drawn with rope traces and marked "U. S.," were quite capable of doing effectual service.

In the field I met a well-dressed, intelligent, Confederate Ambulance Sergeant, who greeted me pleasantly upon my explaining my mission, and who stated that he was there for a like purpose. He had found a few wounded, and they were removed to Savage's Station on the opposite side of the field, where our army, on retiring, had left about 2,000 wounded, chiefly from the Ellerson's and Gaines's Mills' battles. I counted about seventy Union dead, lying in the open space through which the Williamsburg Road emerges into the Savage Station's Field, whose uniforms were mostly new, and whose pockets were inside out indicating that they had been rifled. These dead belonged, for the most part, to Brook's Vermont Brigade. After an hour or so spent in this way upon the field, I returned to my hospital and resumed work. All the weary day long the Confederate column marched down the road leading past our place, and at night some of their trains camped in the woods near us. During the afternoon we heard the firing at White Oak Swamp where a battle was in progress.

July 1. On Tuesday morning we were moved up to Savage's station with our wounded, and merged into that hospital. Dr. John Swinburne, a contract medical officer without rank, had been left in charge by the Medical Director of the Army of the Potomac, and Major Ray, of Magruder's staff, had been designated as Military Commandant by the Confederate authorities. (Note. See transactions of the *Med. Soc. S. N. Y.* for 1863, P. 128, et seq. for Swinburne's report of this hospital.) I remained at Savage's Station two weeks, and assisted in the care of the wounded. The first day, Tuesday, we distinctly heard the guns at Malvern Hill, and soon learned of the terrible punishment that the Army of the Potomac there gave to Lee and his hospital.

I had charge of about 200 wounded at this hospital and made a number of capital operations, besides innumerable minor ones, and assisted at about 100 made by others. The weather was hot and, as a consequence, many of the wounded did badly, from septic causes. The enemy took possession of a considerable part of the rations and stores which were left for us, appropriating them to their own use regardless of our necessities. While at Savage's (no sarcasm intended) a sergeant of the regular army transferred a colored boy, a servant of his Captain, to my custody, who passed as my servant thereafter.

IN RICHMOND.

On Sunday, July 13, we were loaded upon platform cars and moved into Richmond. Arriving there late in the evening, and finding no ambulances in waiting, we remained at the station all night; in the morning we were distributed to the various tobacco warehouses then being used as hospitals. I was assigned quarters in Libby Prison, with the other medical officers. In the afternoon Lieutenant Turner, the commandant of the prison, ordered a search of my person, claiming that he did so to ascertain if I had any counterfeit Confederate money in my possession. I was not the unhappy possessor of that valuable circulating medium, but *did* have some gold, having been paid partly in that coin a few days before; he cast longing eyes at it, but did not dare to take it. It must be remembered that I was a Medical Officer left wounded under proper orders, and not a prisoner of war in the ordinary acceptation of the term. I was soon assigned to the care of wounded in Warehouse No. 4, about four blocks east of "Libby," on Cary Street. The weather was very hot and the stifling air of the building, saturated with the odor of stale tobacco and without any conveniences whatever, was something almost unendurable. It is a wonder that even so many survived as did. I went to Libby for my meals, and also slept there on the bare floor. I met Dr. Joseph W. Robinson on my arrival at Libby, who had been captured at White Oak Swamp. We joined the officers' mess, purchasing our food with the gold and U. S. currency which we had, and which the rebels were very glad to get in exchange for their merchandise of any kind.

July. 17. Thursday. I obtained a pass from Turner, and visited the officers' prison on 18th Street, where Lieutenant McLean and Captain Hamilton were confined, and whose joint guest I became for the night. They were both very glad to see me, and did all in their power to entertain me pleasantly. McLean was cheerful, making the most of everything, and taking his imprisonment as kindly as possible; but Hamilton was the reverse, feeling downcast in the extreme. However, we had a pleasant visit, and the next morning at nine o'clock I returned to Libby. On my arrival there I found that about 500 wounded were being exchanged, and that a part of them had already been loaded into ambulances, which were drawn up in front of the building. I applied to Dr. Cullen, under whose direction the exchange was being made, for permission to go with them. He readily granted my request, and I boarded a convenient ambulance, taking a seat with the driver. The little colored boy, who became separated from me on my arrival at Libby, now came running up with tears in his eyes, begging to go along too. I told him to get aboard, the driver having consented, and we were driven to Aiken's Landing on the James River, about ten miles below Richmond.

On our arrival at the Landing we found the Hospital Steamer "Louisiana" at the wharf awaiting us, and the afternoon was occupied in transferring the wounded to the boat. Lieutenant Colonel Sweitzer of General McClellan's staff was the truce officer in charge, and this was the first transaction under the cartel, just then agreed between the hostile powers. I succeeded in getting the colored boy past the guard and safely on board, through the kindness of the Ambulance Sergeant who was in charge at the wharf, and who was the same whom I had met on the field of Savage's Station on the 30th of June. Dr. Robinson was with me, and we were rejoiced to get under the protection of the Stars and Stripes once more.

(Continued.)

